# Supplementary material for: Small brown planthopper infestation enhances it reproduction and insecticide tolerance by manipulating glucose distribution and levels in rice
Source: eLife. 2026 Oct 26;15:RP110514. doi: 10.7554/eLife.110514 (PMC13707378; doi:10.7554/eLife.110514)
Supplement: Supplementary file 2 [file elife-110514-supp1.docx]

**Supplementary File 1**

**Table A Effects of different treatments on the resistance to imidacloprid in SBPH**

| **treatment** | **LC_50_ (mg/L, 95% CI)** | **slope** |
| --- | --- | --- |
| No Pre-infestation | 2.26 (1.34-3.19) | 1.94±0.24 |
| Pre-infestation | 5.85 (3.68-7.99) | 1.38±0.18 |
| 0.5% glu | 2.35 (1.40-3.31) | 1.96±0.24 |
| 1.0% glu | 5.83 (3.73-7.89) | 1.46±0.19 |
| 1.5% glu | 6.56 (4.29-8.77) | 1.45±0.19 |
| 2.0% glu | 6.46 (4.22-8.58) | 1.50±0.19 |

**Table B Effects of different sugars on the resistance to imidacloprid in SBPH**

| **treatment** | **LC_50_ (mg/L, 95% CI)** | **slope** |
| --- | --- | --- |
| Mannitol | 1.93 (0.97-2.97) | 1.75±0.23 |
| sucrose | 6.17 (3.09-8.50) | 1.38±0.18 |
| sucrose + arabinose | 2.30 (1.40-3.22) | 1.95±0.23 |
| sucrose + arabinose + glu | 6.51 (3.99-9.04) | 1.36±0.19 |
| trehalose | 7.00 (5.26-8.72) | 1.59±0.17 |
| Trehalose + Val A | 1.60 (0.53-2.97) | 1.51±0.23 |
| Trehalose + Val A + glu | 6.12 (3.91-8.28) | 1.47±0.19 |

**Table C Target genes’ names were detected with RT-qPCR in this study**

| **Name of gene** | **Gene bank accession numbers** | **Species** |
| --- | --- | --- |
| ***Vitellogenin* (*LsVg*)** | KC469580.1 | *L. striatellus* |
| ***vitellogenin receptor* (*LsVgR*)** | MH321494.1 | *L. striatellus* |
| ***Target of Rapamycin* (*TOR*)** | QKKF02012754.1 | *L. striatellus* |
| ***juvenile hormone acid methyl transferase* (*JHAMT*)** | MF197905.1 | *L. striatellus* |
| ***Krüppel homolog 1* (*Kr-h1*)** | MK598067.1 | *L. striatellus* |
| ***methoprene-tolerant* (*Met*)** | MK598066.1 | *L. striatellus* |
| ***juvenile hormone esterase* (*JHE*)** | QKKF02024659.1 | *L. striatellus* |
| ***juvenile hormone epoxide hydrolase* (*JHEH*)** | MK598068.1 | *L. striatellus* |
| ***glutathione S-transferase s1 GSTs1*** | JN628440 | *L. striatellus* |
| ***glutathione S-transferase e1(GSTe1)*** | JN628441 | *L. striatellus* |
| ***glutathione S-transferase z1 (GSTz1)*** | JN628442 | *L. striatellus* |
| ***glutathione S-transferase s2 (GSTs2)*** | JN628443 | *L. striatellus* |
| ***glutathione S-transferase s3 (GSTs3)*** | JN628444 | *L. striatellus* |
| ***glutathione S-transferase t1 (GSTt1)*** | JN628445 | *L. striatellus* |
| ***glutathione S-transferase d1 (GSTd1)*** | JN628446 | *L. striatellus* |
| ***glutathione S-transferase m (*GSTm)** | JN628447 | *L. striatellus* |
| ***glutathione S-transferase o1*(*GSTo1*)** | JN628448 | *L. striatellus* |
| ***Glutamate cysteine ligase* (*GCL*)** | cloned in this study | *L. striatellus* |
| ***Isocitrate dehydrogenase* (*IDH*)** | BAS95446.1 | *O. sativa* |
| ***phosphofructokinase* (*PFK*)** | BAS80592.1 | *O. sativa* |

**Table D primers used for qPCR synthesis**

| **Primer name** | **Sequence (5’to3’)** | **Description** |
| --- | --- | --- |
| **qLsVg-F** | CAACATTCTGCCCCAATCCG |  |
| **qLsVg-R** | TTGGCAGCTCATCAACATCGT | *LsVg* qPCR |
| **qLsVgR-F** | TCACGGAAATCAGCGTCTCTAT |  |
| **qLsVgR-R** | AGGATGAACTAAGGCGTGCTC | *LsVgR* qPCR |
| **qTOR-F** | TTGAGCACCGAATCATGCTG |  |
| **qTOR-R** | CATGGACATGACAGCCAGTG | *TOR* qPCR |
| **qJHAMT-F** | AGCAAACTGCAATGGCAAGA |  |
| **qJHAMT-R** | GGCTGTCACATTTCCAGGAC | *JHAMT* qPCR |
| **qKr-h1-F** | AGAGCTGTGCCAATACCTGT |  |
| **qKr-h1-R** | GGCACGGGTGACATTAGAAC | *Kr-h1* qPCR |
| **qMet-F** | AAGGAGGCTACTGGCAACAT |  |
| **qMet-R** | TCTCGATCTCCTTGCAGTCC | *Met* qPCR |
| **qJHE-F** | GACGGCTAACTGATGATCGC |  |
| **qJHE-R** | TGAGCCTTGAACGGAGAACT | *JHE* qPCR |
| **qJHEH-F** | TTTACTGGGTGACAGGCACT |  |
| **qJHEH-R** | CCGTTACACGTGTCAGGTTC | *JHEH* qPCR |
| **qGCL-F** | TACTGCAGTCCTGAACGTGT |  |
| **qGCL-R** | GCACTGACATGCGTCTGAAT | *GCL qPCR* |
| **qGSTs1-F** | TTTTCGCTTATGCTGGTGTTGA |  |
| **qGSTs1-R** | AAAACAGGCAGCATCCCAAA | *GSTs1* *qPCR* |
| **qGSTe1-F** | ACAAATCTTTCCAGGCGTTGA |  |
| **qGSTe1-R** | TCCTTGTTGATTGGCCGAAT | *GSTe1* *qPCR* |
| **qGSTz1-F** | GGGAGATTTGCGAAGTAATTGC |  |
| **qGSTz1-R** | TGCTGTGCCCATTCTTTTTTC | *GSTz1* *qPCR* |
| **qGSTs2-F** | ACCCCATTCGGCCAAGTT |  |
| **qGSTs2-R** | CAGCGATCCCAGCTTTTTTG | *GSTs2* *qPCR* |
| **qGSTs3-F** | ACCGCTCTGGGAGAACCAA |  |
| **qGSTs3-R** | TTGGTTTGATTGATGGCCATT | *GSTs3 qPCR* |
| **qGSTt1-F** | TATCCTTGGAGCCTGCGAAA |  |
| **qGSTt1-R** | TGTTTTTATGCGATTCATCCATTC | *GSTt1 qPCR* |
| **qGSTd1-F** | TTCCCCTACAGCGCTCCAT |  |
| **qGSTd1-R** | GTTGTTTCCCCTCGAAGAGGTT | *GSTd1* *qPCR* |
| **qGSTm-F** | TGGCCGCCCTGCTGTA |  |
| **qGSTm-R** | TTCAGCGCCAACCAATAGG | *GSTm qPCR* |
| **qGSTo1-F** | TCCATTGAGAAAGGCCAAAGA |  |
| **qGSTo1-R** | CGTTTCTGTATCGATGTCGTTGA | *GSTo1* *qPCR* |
| **qARF-F** | TTGGACAGTATCAAGACCCATC |  |
| **qARF-R** | GCAGCAATGTCATCAATAAGC | *ARF* qPCR |
| **qOsIDH-F** | ACCTCAGCACCAAAAACACA |  |
| **qOsIDH-R** | CCCAAACATAGCCTCCTTCA | *OsIDH* qPCR |
| **qOsPFK-F** | TATTTGAGTTTTTGCCCCCA |  |
| **qOsPFK-R** | GCCTTCCTTCATACGCCTGT | *OsPFK* qPCR |
| **OsUBQ5-F** | AAGTAAGGAAGGAGGAGGAAATCG |  |
| **OsUBQ5-R** | AACCAAAAGTAGGGCATCACAATC | *OsUBQ5* qPCR |

**Table E Primers used for dsRNA synthesis**

| **Primer name** | **Sequence (5’to3’)** | **Description** |
| --- | --- | --- |
| **dsGCL-F** | T7TGCAGTAACATGTCCCGGAG |  |
| **dsGCL-R** | T7CGGAAGGTGGCCAGCACTG | dsGCL synthesis |
| **dsTOR-F** | T7ACATCATTCAATCAGGCC |  |
| **dsTOR-R** | T7AACATGTAATCGTTGCCG | dsTOR synthesis |
| **dsJHAMT-F** | T7GATGAGCAAGCTTGCAGAA |  |
| **dsJHAMT-R** | T7GACAAAGTGCCACTACCA | dsJHAMT synthesis |
| **dsKr-h1-F** | T7CTCCGCCCAGCAGCAACCC |  |
| **dsKr-h1-R** | T7TTTTCTAATAATATTAATGA | dsKr-h1 synthesis |
| **dsMet-F** | T7CAACTGCAAAGCGGCCACG |  |
| **dsMet-R** | T7CGTGATGTACTCCTCGCGC | dsMet synthesis |
| **dsGSTe1-F** | T7ATAGAGTCTAGAGGATTC |  |
| **dsGSTe1-R** | T7CACAAAACGAATCCGT | dsGSTe1 synthesis |
| **dsGSTo1-F** | T7CCTCGTTCTAATACATA |  |
| **dsGSTo1-R** | T7AAACTCTCGTAGAGATC | dsGSTo1 synthesis |
| **dsEGFP-F** | T7CATGTATTGGATGAGA |  |
| **dsEGFP-R** | T7CGCATCATGCAGATGT | dsEGFP synthesis |

T7 promoter sequence (5’-TAATACGACTCACTATAGGG-3’)
