# Supplementary material for: Small brown planthopper infestation enhances it reproduction and insecticide tolerance by manipulating glucose distribution and levels in rice
Source: eLife. 2026 Oct 26;15:RP110514. doi: 10.7554/eLife.110514 (PMC13707378; doi:10.7554/eLife.110514)
Supplement: Supplementary file 3 [file elife-110514-supp2.docx]

**Alignment of Glutamate-cysteine ligase (GCL) amino acid sequences**

The sequence of GCL in *Laodelphax striatellus* was compared to those of *Drosophila melanogaster*, *Bombyx mori*, *Caenorhabditis elegans,* and *Homo sapiens,* and the accession numbers of these proteins on NCBI are Q9W3K5.1, BDP27722.1, Q20117.2, and P48506.2 respectively. Blue shadow = glutamate-binding; Pink shadow = magnesium-binding; Purple Frame = ATP/ADP-binding; Green Frame = cysteine-binding; Red Frame = conserved cysteine
