# Supplementary material for: Small brown planthopper infestation enhances it reproduction and insecticide tolerance by manipulating glucose distribution and levels in rice
Source: eLife. 2026 Oct 26;15:RP110514. doi: 10.7554/eLife.110514 (PMC13707378; doi:10.7554/eLife.110514)
Supplement: Peer review history

# Editor report: eLife Assessment

**DOI:** 10.7554/eLife.110514.3.sa0

**Authors:** Sergio Rasmann

**Affiliations:** University of Neuchâtel Switzerland

**Keywords:** Compelling, Important

This **important** study investigates how infestation by the small brown planthopper (Laodelphax striatellus) reshapes rice carbohydrate allocation and demonstrates that host-derived glucose enhances insect fecundity and imidacloprid tolerance, through the activation of conserved nutrient-sensing and endocrine pathways. Across extensive and complementary approaches, including plant manipulations, glucose supplementation, RNAi, pharmacological inhibition, rescue experiments, and biochemical assays, the authors provide strong evidence that glucose activates the TOR-juvenile hormone-vitellogenin axis to promote reproduction and co-regulates GST-mediated detoxification via both TOR-JH signaling and GCL-GSH metabolism. The mechanistic framework is coherent and well supported by hierarchical validation and functional assays. While minor weaknesses exist regarding the generalizability of the findings and the identification of upstream initiating signals, the work provides a **compelling** framework linking nutrient sensing to pest adaptation.

# Referee report: Reviewer #2 (Public review)

**DOI:** 10.7554/eLife.110514.3.sa1

Summary:

Zhang and colleagues investigate the molecular mechanisms by which the small brown planthopper (SBPH, Laodelphax striatellus) manipulates host rice carbohydrate metabolism to enhance its own fitness. Using a combination of molecular, pharmacological, and biochemical approaches, they demonstrate that SBPH infestation induces systemic glucose reallocation in rice, as evidenced by the upregulation of glucose levels in aerial tissues and simultaneous reduction in root glucose levels. Notably, host-derived glucose acts as a central signaling molecule, driving two key adaptive traits: enhanced fecundity via the glucose-TOR-JH-Vg signaling cascade, and increased imidacloprid tolerance through synergistic metabolic (GCL-GSH) and regulatory (TOR-JH-GST) pathways targeting GST activity. These findings uncover a sophisticated resource-manipulation strategy in SBPH and identify nutrient-sensing and detoxification pathways as potential targets for pest control.

Strengths:

(1) The study addresses a gap in plant-insect coevolution research by identifying glucose as a dual-function signaling molecule that coordinates SBPH reproduction and insecticide tolerance, providing valuable insights into how herbivores exploit host nutritional signals.

(2) The experimental design is well structured and multifaceted, integrating RNAi, RT-qPCR, Western blotting, pharmacological inhibition, and biochemical assays. The use of appropriate controls (e.g., osmotic controls with mannitol and hydrolase-inhibitor rescue experiments) strengthens the causal interpretation of the results.

(3) The mechanistic framework is clear and well-supported. The authors delineate two interconnected molecular cascades (glucose-TOR-JH-Vg for fecundity and GCL-GSH/TOR-JH-GST for tolerance) with hierarchical validation (e.g., rescue experiments with JHA), ensuring the reliability of conclusions.

Weaknesses:

(1) The study focuses exclusively on SBPH without validating whether the observed phenomena and mechanisms are conserved in closely related planthopper species (e.g., brown planthopper Nilaparvata lugens). This limitation restricts the generalizability of the findings to other economically important rice pests.

(2) The specific upstream signals that trigger glucose reallocation in rice (e.g., SBPH salivary effectors or oviposition-associated factors) are not identified. Although this represents a complex and independent research direction, the absence of such information limits the depth and completeness of the mechanistic framework and leaves open questions regarding the initiation of host metabolic manipulation.

(3) Insecticide tolerance assays are limited to imidacloprid. Extending these analyses to one or two additional commonly used insecticides (e.g., thiamethoxam) would help determine whether the glucose-mediated detoxification pathway is specific to imidacloprid or reflects a broader resistance mechanism, thereby strengthening conclusions regarding the generality of the GST activation cascade.

(4) Given the study's potential implications for pest management, the manuscript would benefit from a brief discussion of possible practical applications, such as manipulating rice glucose metabolism through breeding strategies or developing small-molecule inhibitors targeting the TOR-JH axis. Including such perspectives would enhance the translational relevance of the work by linking mechanistic insights to real-world pest control strategies.

Comments on revised version.

The authors have comprehensively and satisfactorily addressed all my comments. The revised manuscript shows significant improvement in quality. I have no further questions or suggestions.

# Author comment: Author response

**DOI:** 10.7554/eLife.110514.3.sa2

**Authors:** Hainan Zhang, Qi Zhang, Huichen Ge, Jiaping Wei, Kun Qian, Xiaolong Liu, Hai Li, Jianjun Wang

**Affiliations:** Yangzhou University Yangzhou China; Haimen Agricultural Technology Promotion Center Nantong China; Yangzhou University Yangzhou China; Yangzhou University Yangzhou China; Yangzhou University Yangzhou China; Hubei University Wuhan China; Yangzhou University Yangzhou China; Yangzhou University Yangzhou China

The following is the authors’ response to the original reviews.

**Reviewer #1 (Public review):**

Summary:

The authors investigate how infestation of rice plants by the small brown planthopper (Laodelphax striatellus), an important pest in rice cultivation, alters host plant carbohydrate metabolism and how these changes affect insect physiology and fitness. They show that planthopper infestation leads to a density-dependent increase in glucose levels in rice plants, which the authors suggest results from a redistribution of carbohydrates from roots to shoots. Elevated glucose levels in plants are reflected by increased glucose contents in the insects themselves, an effect that is particularly pronounced in gravid females and associated with enhanced fecundity.

In addition, the authors demonstrate that increased glucose availability enhances tolerance of the small brown planthopper to the neonicotinoid insecticide imidacloprid. These findings suggest that insect-mediated changes in plant carbohydrate allocation may benefit insect fitness in multiple ways, including increased reproductive output and enhanced tolerance to insecticides, both of which are relevant for understanding insect population dynamics in agroecosystems.

Beyond these physiological observations, the authors aim to elucidate the underlying molecular mechanisms. They propose that glucose functions not only as a nutritional resource but also as a signaling molecule. Specifically, they show that increased glucose availability is associated with activation of the Target of Rapamycin (TOR) pathway, a conserved nutrient-sensing signaling pathway regulating growth and metabolism across eukaryotes. Activation of TOR signaling is linked to increased juvenile hormone levels, which in turn stimulate vitellogenesis and likely contribute to increased fecundity. Furthermore, elevated juvenile hormone levels are associated with increased expression of glutathione S-transferases, suggesting a mechanism contributing to enhanced detoxification capacity. Independent of this pathway, increased glucose availability also leads to higher expression of glutamate-cysteine ligase, the rate-limiting enzyme in glutathione synthesis. Together, these mechanisms provide a non-exclusive explanation for the observed increase in imidacloprid tolerance and form the basis of the authors' proposed mechanistic framework linking glucose availability to reproduction and detoxification.

We appreciate the reviewer for the thoughtful and positive summary of our work. We greatly appreciate the careful reading and the constructive recognition of our key findings, including the density‑dependent increase in glucose levels in rice plants, the resulting enhancement of planthopper fecundity, and the link between glucose availability and imidacloprid tolerance.

We are also grateful that the reviewer highlighted our proposed mechanistic model, in which glucose acts as a signaling molecule to activate the TOR pathway, leading to increased juvenile hormone levels, enhanced vitellogenesis, and upregulation of detoxification-related enzymes such as glutathione S‑transferases and glutamate‑cysteine ligase.

We have carefully addressed all other comments from the previous public reviews in the point‑by‑point response below.

Strengths:

A major strength of the manuscript is its substantial mechanistic depth and the extensive use of complementary experimental approaches that converge on a coherent mechanistic interpretation. The authors combine plant manipulations, dietary supplementation, injection assays, RNAi-mediated gene silencing, pharmacological inhibition, and rescue experiments to systematically test the role of glucose as a signaling molecule linking plant-derived nutrition to insect reproduction and insecticide tolerance. Results obtained from independent experimental strategies are highly consistent, and the different datasets collectively support the central conclusions of the study.

The role of glucose is supported by multiple lines of evidence demonstrating that increased glucose availability, whether induced by prior planthopper feeding, dietary supplementation, or direct injection, consistently results in elevated glucose levels in insects, increased oviposition, and enhanced expression of vitellogenesis-related genes (LsVg and LsVgR). The specificity of this effect is further strengthened by experiments using alternative carbohydrates that release glucose upon enzymatic cleavage, as well as inhibitor and rescue experiments, supporting the interpretation that glucose acts beyond a purely nutritional role.

The authors further establish a mechanistic link between glucose availability, TOR signaling, juvenile hormone regulation, and vitellogenesis. Activation of TOR signaling by glucose, demonstrated at the level of protein phosphorylation, together with RNAi knockdown and pharmacological inhibition, allows causal placement of TOR upstream of juvenile hormone signaling. Consistent reductions in juvenile hormone titers, vitellogenesis-related gene expression, and oviposition following TOR inhibition, as well as rescue of reproductive output by juvenile hormone analog treatment, provide strong functional support for a glucose-TOR-juvenile hormone axis regulating fecundity. The absence of additive effects following combined knockdown of TOR and juvenile hormone synthesis components further supports the interpretation that these factors act within the same signaling cascade.

Similarly, the authors provide a detailed mechanistic analysis of glucose-mediated effects on imidacloprid tolerance. Functional assays demonstrate that glutathione S-transferases contribute to detoxification in this species and that increased glucose availability enhances GST activity, glutathione synthesis, and overall glutathione levels. Transcriptomic analyses and targeted RNAi experiments further identify specific GSTs contributing to insecticide tolerance and indicate that glucose enhances detoxification through both TOR-dependent and TOR-independent mechanisms. The combined knockdown experiments, which produce additive effects on mortality, provide particularly strong support for the involvement of multiple interacting glucose-dependent pathways.

We appreciate the reviewer for the highly positive and thorough recognition of our work's strengths, including the mechanistic depth, convergent experimental approaches, and the proposed glucose–TOR–JH signaling cascade.

Weaknesses:

While I am impressed by the mechanistic depth of the study and the clarity with which the authors dissect the underlying physiological pathways, I am less convinced by the current conceptual framing of the phenomenon as a sophisticated adaptive strategy "co-opted" by the small brown planthopper. The data convincingly demonstrate that glucose availability activates conserved nutrient-sensing and endocrine pathways, including TOR signaling and juvenile hormone regulation, which in turn affect reproduction and detoxification capacity. However, these pathways are deeply conserved and likely operate in many insects in response to nutritional status. As such, the results may reflect a general physiological response to elevated carbohydrate availability rather than a species-specific, evolved strategy. Relatedly, herbivory-induced changes in plant carbohydrate allocation appear to be relatively common across plant-insect systems, and it would be helpful to discuss how specific (or general) the observed phenomenon is likely to be.

In particular, I encourage the authors to more clearly distinguish between (i) a conserved nutrient-responsive signaling cascade and (ii) an adaptive mechanism that evolved specifically under selection imposed by insecticide exposure. The presented data strongly support the former interpretation, whereas evidence for the latter is less clear. The increased tolerance to imidacloprid appears to arise as a consequence of enhanced metabolic and detoxification capacity under elevated glucose conditions, rather than as a trait shaped directly by insecticide-driven selection. Framing this phenomenon as an adaptation to insecticide stress may therefore overextend the conclusions that can be drawn from the data. A more cautious discussion acknowledging that glucose-mediated activation of conserved metabolic and endocrine pathways may incidentally enhance insecticide tolerance, without necessarily having evolved under insecticide selection, would strengthen the conceptual clarity of the manuscript.

We fully agree with the concerns raised regarding the evolutionary framing, conceptual definitions. We have thoroughly revised the manuscript to avoid overstatements about adaptive evolution, distinguish between conserved nutrient-responsive pathways and species-specific adaptations, supplement key definitions and literature, and address the study limitations and future directions in Discussion.

While I am impressed by the mechanistic depth of the study and the clarity with which the authors dissect the underlying physiological pathways, I am less convinced by the current conceptual framing of the phenomenon as a sophisticated adaptive strategy "co-opted" by the small brown planthopper.

We appreciate this comment. We replaced “how herbivorous insects exploit host nutritional signals for adaptation” with “how herbivorous insects respond to host nutritional signals to modulate their fitness traits”.

Additionally, we uniformly revised overstated terms such as exploit, co-opt, and adaptive strategy throughout the manuscript to utilize, and nutrient-responsive mechanism, respectively, clarifying that our findings reflect a conserved physiological response of insects to host nutritional signals rather than specialized adaptive evolution under insecticide stress, thus avoiding overstatement of evolutionary adaptation.

The specific revisions are as follows:

(1) “exploit” was revised to “utilize”;

(2) “manipulation” was revised to “change”;

(3) “manipulated resource is exploited” was revised to “nutritional change is utilized”;

(4) The first sentence of the Discussion section “Our study reveals a sophisticated adaptive strategy whereby SBPH actively manipulates host plant carbohydrate metabolism to simultaneously augment its reproductive capacity and insecticide tolerance.” was revised to: “Our study reveals a conserved nutrient-responsive mechanism whereby SBPH infestation elicits a carbohydrate metabolism shift in rice, and the insect subsequently utilizes host-derived glucose to simultaneously augment its reproductive capacity; concurrently, this glucose-mediated pathways enhances insecticide tolerance.”; t

(5) The second sentence of the Discussion section “we identify host-derived glucose as a central resource co-opted by SBPH and delineate two interconnected molecular cascades through which it exerts dual fitness benefits” was revised to: “we identify host-derived glucose as a central signaling molecule that modulates two interconnected molecular cascades exerting dual fitness benefits”.

The data convincingly demonstrate that glucose availability activates conserved nutrient-sensing and endocrine pathways, including TOR signaling and juvenile hormone regulation, which in turn affect reproduction and detoxification capacity. However, these pathways are deeply conserved and likely operate in many insects in response to nutritional status. As such, the results may reflect a general physiological response to elevated carbohydrate availability rather than a species-specific, evolved strategy. Relatedly, herbivory-induced changes in plant carbohydrate allocation appear to be relatively common across plant-insect systems, and it would be helpful to discuss how specific (or general) the observed phenomenon is likely to be.

Thank you for your comments and insights; we fully agree with your perspective. Accordingly, we have made the following revisions in the Abstract, Introduction, and Discussion of our manuscript:

(1) The sentence “Our findings establish host-derived glucose as a central signaling molecule that SBPH exploits to simultaneously optimize reproduction and insecticide resistance.” has been modified to “Our findings establish host-derived glucose as a central signaling molecule that SBPH utilizes to modulate conserved pathways for simultaneous optimization of reproduction and insecticide resistance.”.

(2) We added the following citation in the Introduction: “and sugar-promoted TOR activation has also been reported in *Drosophila* [29]”.

(3) We revised the sentence “However, direct evidence for glucose-mediated TOR activation in insects and its functional connection to JH signaling and reproduction is lacking” by specifying “insects” as “hemipteran insects”.

(4) In the Discussion, we revised “its sensitivity to glucose has remained elusive” to “sugar-promoted TOR activation has been reported in *Drosophila* [29], and our study extends this conserved regulatory mechanism to hemipteran insects”.

(5) We added the phrase “This nutrient-responsive cascade might be conserved across insect species” at the end of the fourth paragraph of the Discussion.

(6) Additionally, we added the following statement in the Discussion: “Notably, studies have shown that brown planthopper (*Nilaparvata lugens*) infestation can reshape sugar distribution in rice by altering the expression of rice sugar transporters, yet the mechanism through which planthoppers regulate these transporters remains unresolved [9]”.

These revisions align with our data and support the reviewer’s view.

In particular, I encourage the authors to more clearly distinguish between (i) a conserved nutrient-responsive signaling cascade and (ii) an adaptive mechanism that evolved specifically under selection imposed by insecticide exposure. The presented data strongly support the former interpretation, whereas evidence for the latter is less clear. The increased tolerance to imidacloprid appears to arise as a consequence of enhanced metabolic and detoxification capacity under elevated glucose conditions, rather than as a trait shaped directly by insecticide-driven selection. Framing this phenomenon as an adaptation to insecticide stress may therefore overextend the conclusions that can be drawn from the data. A more cautious discussion acknowledging that glucose-mediated activation of conserved metabolic and endocrine pathways may incidentally enhance insecticide tolerance, without necessarily having evolved under insecticide selection, would strengthen the conceptual clarity of the manuscript.

We appreciate the professional comments and fully agree with your perspective. Accordingly, we have made the following revisions in the Discussion section:

(1) The sentence “Our study reveals a conserved nutrient-responsive mechanism whereby SBPH infestation elicits a carbohydrate metabolism shift in rice, and the insect subsequently utilizes host-derived glucose to simultaneously augment its reproductive capacity and insecticide tolerance.” has been revised to:

“Our study reveals a conserved nutrient-responsive mechanism whereby SBPH infestation elicits a carbohydrate metabolism shift in rice, and the insect subsequently utilizes host-derived glucose to augment its reproductive capacity; concurrently, this glucose-mediated activation of conserved metabolic pathways incidentally enhances insecticide tolerance.”

(2) Original sentence: “The insect then exploits this manipulated nutritional landscape, deriving dual benefits of increased fecundity and enhanced insecticide tolerance.”

Revised to:

“The insect then exploits this manipulated nutritional landscape to increase fecundity, and the concurrent activation of conserved pathways by glucose incidentally enhances insecticide tolerance.”

To remove the phrase “dual benefits,” which could imply that tolerance is an actively obtained adaptive advantage.

(3) Original sentence:

“Parallel to fecundity enhancement, SBPH utilizes host glucose to bolster its tolerance to the insecticide imidacloprid by supporting a novel dual-pathway model for GST activation, entailing both metabolic fueling and transcriptional regulation.”

Revised to:

“Parallel to fecundity enhancement, the activation of conserved metabolic and endocrine pathways by host-derived glucose incidentally bolsters SBPH tolerance to the insecticide imidacloprid, which is mediated by a novel dual-pathway model for GST activation involving both metabolic fueling and transcriptional regulation.”

To clarify that the enhancement of insecticide tolerance is an incidental consequence of pathway activation, not a direct utilization strategy.

**Reviewer #1 (Recommendations for the authors):**

(1) Line 26 (Abstract): "how herbivorous insects exploit host nutritional signals for adaptation remains unclear." I am not sure that what is described here constitutes exploitation of a signal for adaptation. The authors convincingly unravel mechanisms by which insects benefit from elevated glucose, but the wording implies an evolved adaptation to insecticide pressure. Given that herbivore effects on nutrient allocation are likely widespread, I would recommend more cautious phrasing and clearer separation between physiological mechanisms and evolutionary interpretations.

We appreciate this comment. We replaced “how herbivorous insects exploit host nutritional signals for adaptation” with “how herbivorous insects respond to host nutritional signals to modulate their fitness traits”.

Additionally, we uniformly revised overstated terms such as exploit, co-opt, and adaptive strategy throughout the manuscript to utilize, and nutrient-responsive mechanism, respectively, clarifying that our findings reflect a conserved physiological response of insects to host nutritional signals rather than specialized adaptive evolution under insecticide stress, thus avoiding overstatement of evolutionary adaptation.

The specific revisions are as follows:

(1) “exploit” was revised to “utilize”;

(2) “manipulation” was revised to “change”;

(3) “manipulated resource is exploited” was revised to “nutritional change is utilized”;

(4) The first sentence of the Discussion section “Our study reveals a sophisticated adaptive strategy whereby SBPH actively manipulates host plant carbohydrate metabolism to simultaneously augment its reproductive capacity and insecticide tolerance.” was revised to: “Our study reveals a conserved nutrient-responsive mechanism whereby SBPH infestation elicits a carbohydrate metabolism shift in rice, and the insect subsequently utilizes host-derived glucose to simultaneously augment its reproductive capacity; concurrently, this glucose-mediated pathways enhances insecticide tolerance.”;

(5) The second sentence of the Discussion section “we identify host-derived glucose as a central resource co-opted by SBPH and delineate two interconnected molecular cascades through which it exerts dual fitness benefits” was revised to: “we identify host-derived glucose as a central signaling molecule that modulates two interconnected molecular cascades exerting dual fitness benefits”;

(6) The phrase “This nutrient-responsive cascade might be conserved across insect species” was added at the end of the fourth paragraph in the Discussion section;

(2) Line 37 (Abstract): To improve readability, please define "LsGST" on first use

We appreciate this comment and have added taxonomic definitions for *LsGSTe1* and *LsGSTo1* at their first appearance in the Abstract: “*LsGSTe1* (SBPH epsilon class GST) and *LsGSTo1* (SBPH omega class GST)”.

(3) Lines 38-39 (Abstract): The repeated framing as "signal exploitation" may not be fully justified, since glucose is simultaneously a key energetic resource that could plausibly fuel parts of the observed response. Clarifying what is meant by "signal" versus "resource" in this context would improve conceptual clarity.

We appreciate this comment. Following your suggestion, we revised the description related to “signal exploitation”, and the sentence “Our findings establish host-derived glucose as a central signaling molecule that SBPH exploits to simultaneously optimize reproduction and insecticide resistance.” has been modified to “Our findings establish host-derived glucose as a central signaling molecule that SBPH utilizes to modulate conserved pathways for simultaneous optimization of reproduction and insecticide resistance.”.

In addition, we have emphasized the signaling role of glucose in both the Results and Discussion sections. Through mannitol osmotic control treatments, hydrolase inhibition assays, and rescue experiments, we excluded the possibility that glucose acts merely as an energy source and confirmed its signaling function in regulating the JH pathway via TOR phosphorylation. These experiments clearly distinguish its signaling role from its nutritional/energetic role.

(4) Lines 39-41 (Abstract): The phrase "nutrient-based control strategies" is difficult to interpret without at least a brief example or explanation. A short clarification would help readers understand the applied implications.

We fully agree with and appreciate this comment. We added a concrete example in the Abstract: “, such as disrupting insect nutrient-sensing pathways or modulating host carbohydrate metabolism”.

We also added a new section “The identification of the glucose‑TOR‑JH axis as a key regulator of SBPH fecundity and insecticide tolerance provides novel strategies for eco-friendly, nutrient-based pest control. Firstly, varieties that limit SBPH-induced glucose redistribution would reduce reproduction and insecticide tolerance without yield loss. Secondly, small-molecule inhibitors targeting TOR phosphorylation or JH synthesis can serve as biopesticides or synergists to improve insecticide efficacy, as they would suppress the glucose-mediated incidental enhancement of insecticide tolerance. Finally, optimized fertilization and irrigation can reduce shoot glucose accumulation and suppress SBPH outbreaks. These strategies offer sustainable alternatives to traditional insecticides and help mitigate insecticide resistance of SBPH.” in the Discussion, detailing three practical strategies (rice breeding, small-molecule inhibitor, agronomic management) to clarify the applied meaning of nutrient-based control strategies.

(5) Line 68 (Introduction): The statement that glucose is "the dominant transportable carbon source" in plants seems inaccurate; sucrose is generally considered the main transport sugar. Consider revising.

We appreciate this professional comment. According to the literature, glucose can be transported in plants but is not the primary sugar involved; sucrose is the main transported form. We have therefore removed the word “dominant”, and the revised description is consistent with current knowledge.

(6) Line 82 (Introduction): The claim that "direct evidence for glucose-mediated TOR activation in insects...is lacking" may not be correct. For example, Kim & Neufeld (2015) report sugar-promoted TOR activation in Drosophila (Nat. Commun., doi: 10.1038/ncomms7846). This may also relate to statements later in the manuscript (e.g., around line 506).

We apologize for this oversight during our initial literature review and sincerely appreciate this professional comment. We have added the relevant citation in the Introduction: “and sugar-promoted TOR activation has also been reported in *Drosophila* [29]”. (of the revised manuscript)

Furthermore, we revised the sentence “However, direct evidence for glucose-mediated TOR activation in insects and its functional connection to JH signaling and reproduction is lacking” by specifying “insects” as “hemipteran insects”. (of the revised manuscript)

In addition, we modified the corresponding statement in the Discussion section: the phrase “its sensitivity to glucose has remained elusive” was revised to “sugar-promoted TOR activation has been reported in *Drosophila* [29], and our study extends this conserved regulatory mechanism to hemipteran insects”. (of the revised manuscript)

These revisions could clarify that our innovative contribution lies in extending this conserved mechanism from *Drosophila* to hemipteran insects, rather than reporting the first discovery of glucose-induced TOR activation. Accordingly, we have adjusted the reference numbering for all subsequent citations in the manuscript.

(7) Line 176 (and elsewhere): Mannitol is used as an osmotic control; it would be helpful to briefly explain why osmolarity is expected to be a relevant confound in these assays and how osmotic effects might otherwise influence the measured outcomes.

We greatly appreciate this valuable comment. We have added the following paragraph to the Discussion section: “Given that osmotic pressure, a key determinant of plant cell turgor pressure, can disrupt insect homeostasis and impair fitness when insects ingest hyperosmotic plant sap [47,48], we rigorously excluded confounding effects of rice osmotic pressure in this study.”.

Two relevant references [47, 48] have been cited to support this statement, and we have adjusted the reference numbering for all subsequent citations in the manuscript.

(8) Line 464 ff.: The statement that co-option of plant defenses by insects is an "emerging paradigm" seems overstated; classic examples such as sequestration of plant toxins have been known for decades. A more nuanced phrasing may be appropriate.

We appreciate this comment and agree with your perspective. We have revised “emerging paradigm” to “classic paradigm” for greater objectivity.

(9) Lines 491-492: This passage is somewhat confusing with respect to framing: here, elevated glucose is described as a plant stress response, whereas elsewhere (including title/abstract) it is presented as manipulation by the insect. Clarifying whether the authors view elevated glucose primarily as a plant response that insects benefit from, versus an actively induced manipulation, would improve consistency.

We greatly appreciate your professional comment. We have revised the relevant statement from: “Given that elevated sugar levels might enhance plant stress resistance [46,47], our study reveals an intriguing ecological paradox: the plant's potential attempt to mount a stress response via glucose accumulation is effectively co-opted by the insect to enhance its own fitness and resilience.”

To: “Notably, studies have shown that brown planthopper (*Nilaparvata lugens*) infestation can reshape sugar distribution in rice by altering the expression of rice sugar transporters, yet the mechanism through which planthoppers regulate these transporters remains unresolved [9]. Given that elevated sugar levels might enhance plant stress resistance [49,50], our study reveals an intriguing ecological paradox that SBPH infestation likely manipulates glucose distribution via unidentified pathways to boost its own fitness and resilience.”

(10) Discussion (general): In addition to the demonstrated glutathione/GST mechanisms, elevated glucose could plausibly support detoxification in other ways (e.g., providing a substrate for conjugation in phase II metabolism). It may be worth briefly acknowledging such additional routes, even if not tested here.

We appreciate this comment. We added the following text in the GST pathway section of the Discussion: “Beyond the GCL-GSH-GST and TOR-JH-GST pathways characterized in this study, elevated glucose may also enhance insecticide detoxification through additional routes (e.g., providing carbon skeletons for phase II xenobiotic conjugation reactions or fueling energy-dependent detoxification processes in insect midgut and fat body), which warrant further experimental verification.”, objectively acknowledging other potential pathways and listing them as future research directions.

**Reviewer #2 (Public review):**

Summary:

Zhang and colleagues investigate the molecular mechanisms by which the small brown planthopper (SBPH, Laodelphax striatellus) manipulates host rice carbohydrate metabolism to enhance its own fitness. Using a combination of molecular, pharmacological, and biochemical approaches, they demonstrate that SBPH infestation induces systemic glucose reallocation in rice, as evidenced by the upregulation of glucose levels in aerial tissues and a simultaneous reduction in root glucose levels. Notably, host-derived glucose acts as a central signaling molecule, driving two key adaptive traits: enhanced fecundity via the glucose-TOR-JH-Vg signaling cascade, and increased imidacloprid tolerance through synergistic metabolic (GCL-GSH) and regulatory (TOR-JH-GST) pathways targeting GST activity. These findings uncover a sophisticated resource-manipulation strategy in SBPH and identify nutrient-sensing and detoxification pathways as potential targets for pest control.

Strengths:

(1) The study addresses a gap in plant-insect coevolution research by identifying glucose as a dual-function signaling molecule that coordinates SBPH reproduction and insecticide tolerance, providing valuable insights into how herbivores exploit host nutritional signals.

(2) The experimental design is well structured and multifaceted, integrating RNAi, RT-qPCR, Western blotting, pharmacological inhibition, and biochemical assays. The use of appropriate controls (e.g., osmotic controls with mannitol and hydrolase-inhibitor rescue experiments) strengthens the causal interpretation of the results.

(3) The mechanistic framework is clear and well-supported. The authors delineate two interconnected molecular cascades (glucose-TOR-JH-Vg for fecundity and GCL-GSH/TOR-JH-GST for tolerance) with hierarchical validation (e.g., rescue experiments with JHA), ensuring the reliability of conclusions.

We thank the reviewer for recognizing the novelty of the scientific question, rigor of the experimental design, and clarity of the mechanistic framework in our study. We fully agree with the limitations raised regarding the generality of the findings, identification of upstream signals, range of insecticides tested, and translational applications for pest management. We have supplemented the manuscript with discussions of our study limitations and future research directions, added a section on the application of our findings in pest control, and provided key future directions such as identification of upstream signal identification, validation of generality and expansion of insecticide testing.

Weaknesses:

(1) The study focuses exclusively on SBPH without validating whether the observed phenomena and mechanisms are conserved in closely related planthopper species (e.g., brown planthopper Nilaparvata lugens). This limitation restricts the generalizability of the findings to other economically important rice pests.

We appreciate this valuable comment. We have added a subsection titled “Limitations and Future Research Directions” in the Discussion section, explicitly stating that this study focuses exclusively on SBPH and the broader generality of the mechanism remains to be verified. Among the future directions outlined, “verifying the conservation of the glucose‑TOR‑JH axis in other economically important rice planthoppers” is designated as the first key research priority, and cross‑species validation experiments are planned accordingly.

(2) The specific upstream signals that trigger glucose reallocation in rice (e.g., SBPH salivary effectors or oviposition-associated factors) are not identified. Although this represents a complex and independent research direction, the absence of such information limits the depth and completeness of the mechanistic framework and leaves open questions regarding the initiation of host metabolic manipulation.

We greatly appreciate this insightful comment. We have incorporated this issue as a key future research direction in the Discussion section. Specifically, we added the following statement: “Notably, the upstream signals (e.g., specific salivary effectors secreted by SBPH or oviposition-associated plant response factors) that trigger glucose reallocation in rice remain uncharacterized and represent a key direction for future in-depth research.”

In addition, we have added a subsection titled “Limitations and Future Research Directions” in the Discussion, which includes the third point: “(3) Identifying the specific SBPH salivary effectors and plant signaling pathways that trigger glucose reallocation in rice, to complete the mechanistic framework of host metabolic changes manipulated by herbivores.”

(3) Insecticide tolerance assays are limited to imidacloprid. Extending these analyses to one or two additional commonly used insecticides (e.g., thiamethoxam) would help determine whether the glucose-mediated detoxification pathway is specific to imidacloprid or reflects a broader resistance mechanism, thereby strengthening conclusions regarding the generality of the GST activation cascade.

We greatly appreciate this comment. Related discussion was added in the subsection titled “Limitations and Future Research Directions” as following:

Expanding assays to other commonly used rice insecticides (e.g., thiamethoxam, pymetrozine, triflumezopyrim) to validate whether the glucose-mediated detoxification pathway confers broad-spectrum tolerance.

(4) Given the study's potential implications for pest management, the manuscript would benefit from a brief discussion of possible practical applications, such as manipulating rice glucose metabolism through breeding strategies or developing small-molecule inhibitors targeting the TOR-JH axis. Including such perspectives would enhance the translational relevance of the work by linking mechanistic insights to real-world pest control strategies.

We greatly appreciate your professional comment. We have added a standalone paragraph in the Discussion section to discuss the novel strategies for SBPH control provided by this study, as follows: “The identification of the glucose‑TOR‑JH axis as a key regulator of SBPH fecundity and insecticide tolerance provides novel strategies for eco-friendly, nutrient-based pest control. Firstly, varieties that limit SBPH-induced glucose redistribution would reduce reproduction and insecticide tolerance without yield loss. Secondly, small-molecule inhibitors targeting TOR phosphorylation or JH synthesis can serve as biopesticides or synergists to improve insecticide efficacy, as they would suppress the glucose-mediated incidental enhancement of insecticide tolerance. Finally, optimized fertilization and irrigation can reduce shoot glucose accumulation and suppress SBPH outbreaks. These strategies offer sustainable alternatives to traditional insecticides and help mitigate insecticide resistance of SBPH.”
